# Bilayer synergetic coupling double negative acoustic metasurface and cloak

**DOI:** 10.1038/s41598-018-24231-3

**Published:** 2018-04-12

**Authors:** Fuyin Ma, Meng Huang, Yicai Xu, Jiu Hui Wu

**Affiliations:** 1 School of Mechanical Engineering & State Key Laboratory for Strength and Vibration of Mechanical Structure, Xi’an Jiaotong University, Xi’an, 710049 China; 2 State Key Laboratory of Explosion Science and Technology, Beijing Institute of Technology, Beijing, 100081 China

## Abstract

In this paper, we propose a bilayer plate-type lightweight double negative metasurface based on a new synergetic coupling design concept, by which the perfect absorption, double negative bands, free manipulation of phase shifts with a 2π span and acoustic cloak can be successively realized. Firstly, the synergetic behavior between resonant and anti-resonant plates is presented to construct a bilayer unit in which each component respectively provides a pre-defined function in realizing the perfect absorption. Based on this bilayer structure, a double negative band with simultaneously negative effective mass density and bulk modulus is obtained, which, as a metasurface, can obtain continuous phase shifts almost completely covering a 2π range, thus facilitating the design of a three-dimensional (3D) acoustic cloak. In addition, based on this strong sound absorption concept, a two-dimensional (2D) omnidirectional broadband acoustical dark skin, covering between 800 to 6000 Hz, is also demonstrated through the proposed bilayer plate-type structure form. The proposed design concepts and metasurfaces have widespread potential application values in strong sound attenuation, filtering, superlens, imaging, cloak, and extraordinary wave steering, in which the attributes of strong absorption, double negative parameters or continuous phase shifts with full 2π span are required to realize the expected extraordinary physical features.

## Introduction

Acoustic metamaterials are some types of man-made composite medium structured on a scale much smaller than a wavelength and developed from the locally resonant structure with negative effective mass density^1^. Besides this negative effective mass density, other effective parameters like the effective negative bulk modulus^2,3^, Poisson’s ratio^4^, shear modulus^5^, and moment of inertia^6^ have also been demonstrated in previous research studies. In addition to lots of single negative structures, several composite devices with double negative parameters have also been achieved by combining two kinds of units comprising different single negative parameters^3–11^. Notably, Yang *et al*. proposed a bilayer membrane-type acoustic metamaterial, in which two membranes were coupled with sealed air and a rigid ring, and achieved a double-negative band both in theory and in experiment^12^. Moreover, other studies have shown that simultaneously double negative parameters can be achieved by folding a geometrically-induced band through labyrinth structures^13,14^, while one recent work realized a double negative band by considering the multiple scattering effect into a locally resonant system with the single negative parameter^15^.

The focus, in recent years, has been to manipulate sound waves within the thinnest structures and smallest possible spaces, as well as to achieve arbitrary control of the amplitudes and phases of the wavefront. Metasurfaces that are deep subwavelength wavefront-shaping devices have been developed from metamaterials to provide the strategies required for this sound wave manipulation^16^. Specifically, the design of an acoustic metasurface array with continuous phase shifts fully covering a 2π range is expected to achieve an unconventional acoustic steering capability. In 2013, Li *et al*. constructed an acoustic metasurface for controlling reflection waves based on a labyrinth structure, and further realized the acoustic superlens^17^; subsequently, Zhao *et al*. proposed a sound wavefront manipulation method using non-uniform impedances and tunable abnormal reflections, thereby realizing the discontinuous surface impedances and sound wave reorientation through a Helmholtz resonator^18,19^. In 2014, Ma *et al*. experimentally achieved a perfect sound absorption in low frequency ranges by employing a membranous structure with a back cavity^20^; Cai *et al*. constructed a labyrinth type acoustic metasurface by 3D printing, and further experimentally realized the perfect sound absorption^21^; while Xie *et al*. realized wavefront modulation and subwavelength diffraction through a labyrinth type acoustic metasurface^22^. In addition to these works, a few studies have explored the application of acoustic metasurfaces in the development of the acoustic cloak^23–26^. These works suggested that both the labyrinth- and membrane-type structures are expected to achieve perfect sound absorption^20,21,27^ and that, to realize this, both the impedance perfectly matching to the air and a cavity surrounded by rigid reflection walls are usually required.

This paper proposes a bilayer plate-type structure based on a new synergetic coupling design concept, which is assembled with one resonant front plate and one anti-resonant back plate. The achievement of perfect sound absorption^20,21,27,28^ is demonstrated both in theory and experiment. Since the structures are similar to previous coupled bilayer membrane-type double negative acoustic metamaterials, the simultaneously double negative parameters are also achieved. Moreover, as the formation of an air cavity is required^20^, metasurfaces with continuous phase shifts covering a 2π range are obtained by adjusting the single dimensional structural or material parameters, with the subsequent successive realization of, first, a 3D acoustic cloak with a narrow band performance and, thereafter, a 2D omnidirectional broadband acoustical dark skin. The results confirm that the proposed bilayer structure offers the capacity to create innovative acoustical functions with sound absorption, double negative effective parameters, continuous phase shifts covering a 2π span, and cloak performances.

## Bilayer Synergetic Coupling Unit with Perfect Absorption

**Figure 1:**
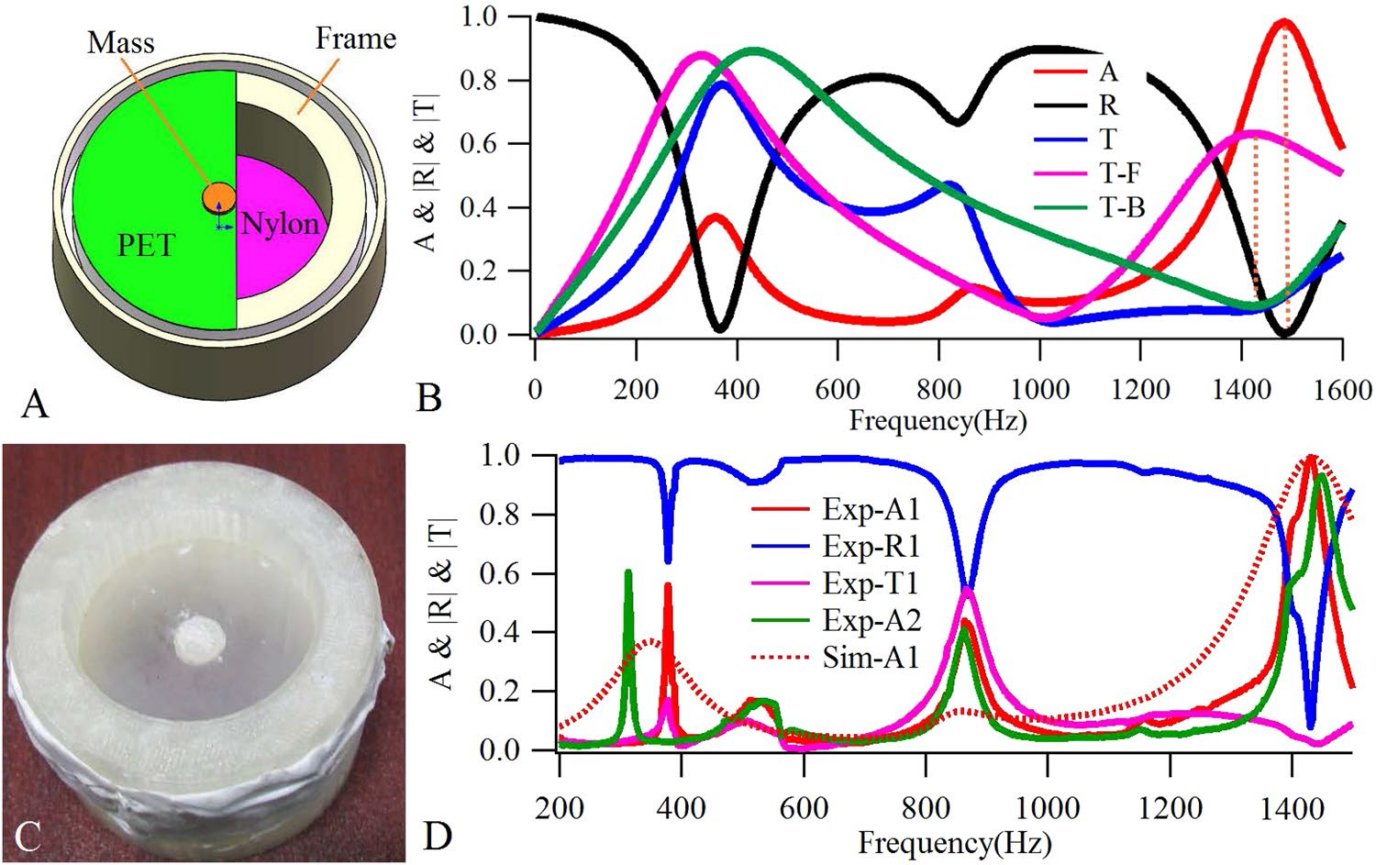
Calculated and measured results of the bilayer plate-type structures. (**A**) The diagram of the designed bilayer plate-type unit structure. (**B**) The calculated sound absorptions (A), reflections (R), transmissions (T) of the assembled unit and the transmissions of back (T-B) and front (T-F) layers, respectively. (**C**) The fabricated bilayer plate-type sample with 100 mg additional mass for measurement. (**D**) The experimentally measured sound absorptions (Exp-A1 and Exp-A2, for samples with 100 mg and 200 mg additional masses, respectively), reflections (Exp-R1), transmissions (Exp-T1) and the numerically fitted sound absorptions (Sim-A1) for the structure with 100 mg additional mass in experiment.

The membrane-type metasurface unit previously proposed^20^ can be considered as a synergetic coupling device consisting of three parts: a membrane-mass unit with in-phase resonances to the incident wave, a total reflection rigid back wall, and an air cavity surrounded by reflection walls and membrane. In this study, the rigid back wall was replaced by an anti-resonant acoustic metamaterial unit with strong reflections at the designed frequencies^29^. Using a bilayer plate-type structure as an example, the design of such a synergetic coupling system includes the following steps: Firstly, we designed a front plate unit to realize the in-phase resonances with incident waves. Next, we designed a back plate unit to produce a strong reflection (always at the anti-resonance frequencies). Thereafter, we assembled the two above-mentioned parts separated at a certain distance by a rigid frame to form an air cavity between the layers. Lastly, we adjusted the resonance frequency of the front plate and an anti-resonance frequency of the back plate to the same position. Figure 1A shows the designed structure (model-1), the sound absorptions (A), reflections (R) and transmissions (T for the assembly structure, T-B and T-F for the back and front plates, respectively), which are solved by the finite element method and plotted in Fig. 1B. The Young’s modulus, Poisson’s ratio and mass density of the back plate are (3.1 + 0.31i) GPa, 0.28, and 1000 kg/m^3^, respectively, and those of the front plate are (2.3 + 0.23i) GPa, 0.37, and 1000 kg/m^3^, respectively. The imaginary part of the Young’s modulus represents the viscoelastic damping loss of the materials (damping ratio is 10%). The evidence in Fig. 1B suggests that at 1426 Hz, transmissions of the back plate (T-B) reach a dip, while those of the front plate (T-F) reach a peak, thereby indicating that the back plate becomes anti-resonant and the front plate becomes resonant with incident waves. Furthermore, from the absorptions (A), reflections (R) and transmissions (T) of the assembly unit, the appearance of a strong absorption peak with an amplitude of 0.98 and a reflection dip can be seen at 1484 Hz. Compared with the transmission peak/dip of the single layer corresponding to the first orange vertical dashed line, the sound absorption peak frequency of the bilayer composite structure, denoted by the second orange vertical dashed line, is higher than the second transmission peak of T-F, due to the vibration of the whole structure as a result of the hybrid resonance of the two plates and the air cavity, as well as the additional impedance to the vibration plates that could be applied by the sealed air^20^. The overall effect of the synergetic coupling behavior is thus clearly demonstrated, as shown in Fig. 1.

The designed bilayer units, one of which is shown in Fig. 1C, were fabricated and their absorptions (Exp-A1 and Exp-A2), reflections (Exp-R1) and transmissions (Exp-T1) were measured by a B&K-4206 impedance tube system. The calculation results of the absorptions were then compared with the experiment results, plotted in Fig. 1D. It was found that the front plate used in the experiment did not perfectly match the material parameters in the preceding calculations, resulting in a new numerical simulation result (Sim-A1) obtained from mechanical properties of the experiment, for which the Young’s modulus of the front plate is (2.08 + 0.208i) GPa. The other parameters were found to be the same as those used in Fig. 1B. From Fig. 1D it can be seen that the measurement and numerical fitting of the absorptions (Exp-A1 and Sim-A1) are in agreement with each other, both in the frequencies at each of the three obvious peaks and the amplitudes at the third peak with strong absorption. The subtle deviation between the measured and calculated results (modle-2), especially in amplitudes at the first and second absorption peaks, may be caused by errors in fabrication and the nonlinearity of material parameters (especially the damping) in the actual sample. It is significant that at the third absorption peak (1430 Hz and 1432 Hz for Exp-A1 and Sim-A1, respectively), the measured result reached up to 0.993 (0.991 for Sim-A1), experimentally demonstrating a perfect absorption. At this frequency, Exp-R1 and Exp-T1 reached the minimum values of 0.079 and 0.022, respectively. In addition, to investigate the tunable property of the absorptions, another sample was fabricated with the additional mass increasing from 100 mg to 200 mg. The sound absorptions for this unit were measured and are plotted in Fig. 1D (Exp-A2). The comparison between the results of Exp-A1 and Exp-A2 concludes that the increased mass in the second unit had the following effects: the frequency of the first peak is the blue shift (377 Hz and 313 Hz for Exp-A1 and Exp-A2, respectively), and that of the third peak is the red shift (1430 Hz and 1447 Hz for Exp-A1 and Exp-A2, respectively), while that of the second peak remained almost unchanged (867 Hz and 864 Hz for Exp-A1 and Exp-A2, respectively). These results suggest that the strong absorption appearing at the peak frequency is tunable, and that the absorption coefficient can be optimized for the perfect absorption. The calculation shows that the sound absorption coefficient could be kept above 0.9 in the range from approximately 1280 to 1560 Hz (corresponding to the Young’s modulus of the front plate from 1.4 to 2.7 GPa). Furthermore, the absorption coefficient at the third peak in both experiments was also reduced to 0.93.

**Figure 2:**
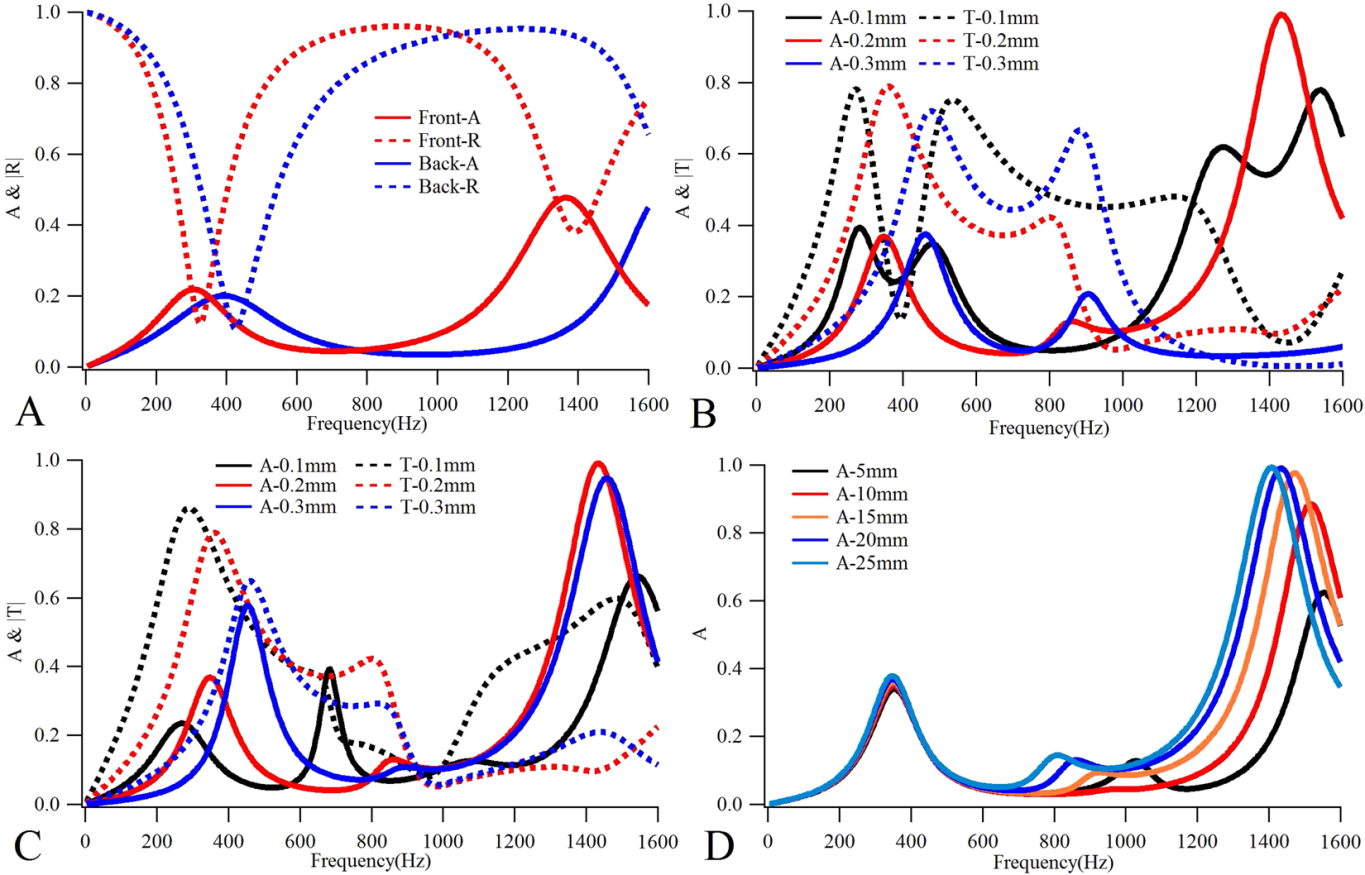
Calculated results of the single layer and the bilayer structures under different parameters. (**A**) The sound absorptions (Front-A and Back-A) and reflections (Front-R and Back-R) of the single front and back layer structures. The sound absorptions and transmissions of the bilayer structures with different front (**B**) and back (**C**) plate thicknesses. (**D**) The sound absorptions of the bilayer structures with different thickness of air layers.

In a clear analysis of the physical principle of the absorption, as plotted in Fig. 2A, the sound absorptions of the single front and back plates suggest that the front plate produced two absorption peaks at 304 Hz and 1368 Hz, respectively, while the back plate produced one absorption peak at 392 Hz. The curve trend of back-A suggests that the second absorption peak would have been produced at some frequency slightly higher than 1600 Hz. The maximum absorptions of both front-A and back-A are lower than 0.5. A comparison between the absorption coefficient curves of the single plates, in Fig. 2A, and of the bilayer structure, in Fig. 1B, shows that the first (second) absorption peak of the bilayer structure is located between the first (second) absorption peaks of the front and the back plates, and that the amplitude of the absorption peak of the bilayer structure is almost equivalent to the superposition of the corresponding front and back plate peaks. Therefore, on the one hand, the sound absorption effect of such a bilayer structure is a superposition of absorptions of the front and the back plates, which is different from that of the single layer membrane structures with a back cavity, as cited in the literature^20^; on the other hand, as no absorption peak is produced by the single layer structure at a frequency near the second absorption peak of the bilayer structure, the amplitude of the second peak is small. In addition, as Fig. 2B and C show, the sound absorption and transmission coefficients of the bilayer structure have different thicknesses on the front and the back plates, which suggests that by changing the thickness of the front or the back plate, not only will the absorption peak frequencies move, but the sound absorption coefficient will decrease as well. Moreover, the absorptions of the structures with different thickness of air layers, as plotted in Fig. 2D, suggest that increasing the thickness from 5 mm to 20 mm, will gradually increase the absorption value at the third peak. If the thickness is further increased to 25 mm, the sound absorption coefficient tends to reach saturation, followed by perfect sound absorption.

Next, we put forward a general design principle for the proposed synergetic coupling design concept that takes into consideration the acoustic reflection properties of the plate. This bilayer synergetic plate-type structure requires the following specific conditions in order to realize the perfect sound absorption: With ${R}_{f}$ as the reflection coefficient of the single front plate and ${R}_{b}$ as that of the single back plate, the reflection coefficients should satisfy the condition that ${R}_{f}$ = 0 and ${R}_{b}$ = 1 at a design frequency of ${f}_{1}$. That is, at the design frequency, the impedance of the front plate should perfectly match that of the sound propagation media (in this case, air) without any reflection (${R}_{f}$ = 0), while the back plate should produce anti-resonances with the incident sound wave for total reflection (${R}_{b}$ = 1). Due to the hybrid resonance of the combined structure, its working frequency, ${f}_{2}$, should be higher than the design frequency of ${f}_{1}$ that is determined by the single layer plates. In fact, hybrid resonances will not only affect the working frequency of the whole structure, but will also produce an additional effective reflection coefficient ${R}_{a}$. If the thickness of the intermediate air layer *d* is small, ${R}_{a}$ will become higher, which will reduce the sound absorption performance of the whole structure. Therefore, in order to realize perfect absorption, the intermediate air layer should have a certain minimum thickness to ensure ${R}_{a}$ = 0. As can be seen from Fig. 2D, the minimum thickness here is approximately 20 mm. In addition, it is necessary for the acoustic wave to be incidental from the impedance matched front plate, because if it is incidental from the back plate, the reflection coefficient of the whole structure will be 1, and the sound absorption coefficient will be 0, which is not the expected result. In summary, in order to realize the perfect absorption all of the following conditions are essential: a) ${R}_{f}$ = 0; b) ${R}_{b}$ = 1; c) ${R}_{a}$ = 0; and d) the sound wave is incident from the impedance matched plate. Because only the resonant and anti-resonant properties of the plate are used in the synergistic coupling design, other physical parameters and properties are not restricted and this design concept can, therefore, be applicable to different shapes of plates, membranes or other types of structures.

## Acoustic Metasurface with Simultaneously Negative Density and Modulus

The dynamic effective parameters are critical to the success of the acoustic metamaterials. Therefore, in this section, the effective mass density and bulk modulus of the above-mentioned model-1 (used in Fig. 1B) will be investigated, based on effective media theories^30,31^. By definition, the effective mass density is

1 $${\rho }_{eff}=\langle {F}_{z}\rangle /\langle \ddot{w}\rangle $$

where $\langle {F}_{z}\rangle$ is the area-averaged force applied on the sample in the z-direction. For a circular plate it reads:

2 $$\langle {F}_{z}\rangle ={{\int }_{0}^{2\pi }{\int }_{0}^{D/2}Pdrd\varphi |}_{z=0}-{{\int }_{0}^{2\pi }{\int }_{0}^{D/2}Pdrd\varphi |}_{z=H}$$

where $P$ is the externally applied pressure, $\langle \ddot{w}\rangle$ is the area-averaged acceleration in the z-direction, and the sound wave incident from the plate side of $z=0$.

**Figure 3:**
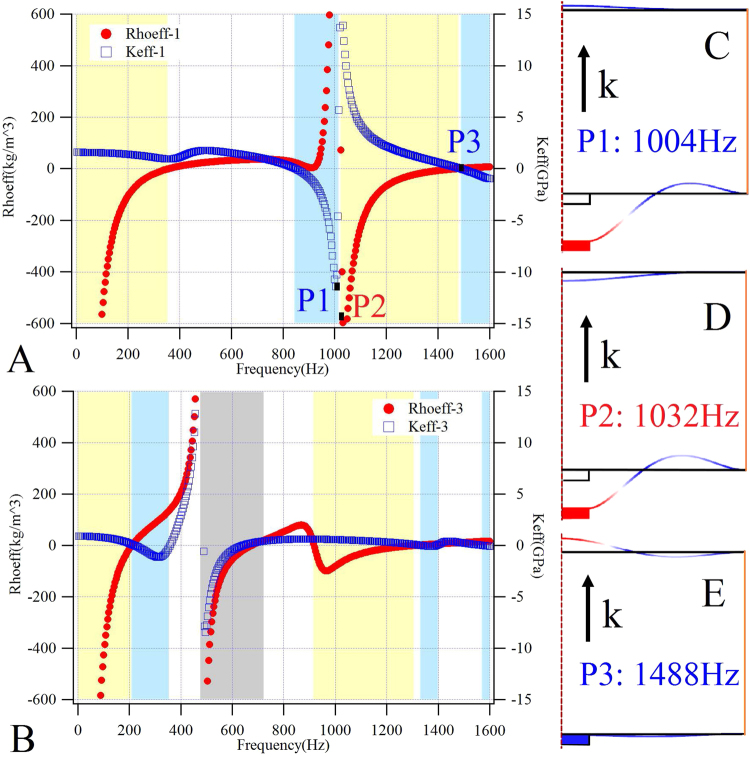
The effective parameters and vibration displacement contours. The calculated ${\rho }_{eff}$ (red curves) and ${K}_{eff}$ (blue curves) of the model-1 (**A**) and model-3 (**B**), wherein the yellow (cyan) shaded areas represent the negative ${\rho }_{eff}$ (${K}_{eff}$) bands, the gray shaded area represents the double negative band, and the damping ratio is reduced to 1%. The vibration displacement contours at point-P1 (**C**), point-P2 (**D**), and point-P3 (**E**). In (**C**) to (**E**), with red/blue representing the maximum/minimum displacement respectively, black arrows the sound incident direction, and the black frames the equilibrium position of plates and masses.

The red solid circle marked curve in Fig. 3A denotes the calculated effective mass density results. The effective bulk modulus was determined using the following formula:

3 $${K}_{eff}=\langle {\sigma }_{b}\rangle /\langle {\varepsilon }_{b}\rangle =-V\langle {T}_{Z}^{c}\rangle /{\rm{\Delta }}V$$

where $\langle {\sigma }_{b}\rangle$ is the area-averaged bulk stress of the plate, $\langle {\varepsilon }_{b}\rangle$ is the averaged bulk strain of the system, *V* is the static volume of the sealed air, $\langle {T}_{Z}^{c}\rangle$ is the normal compressional stress averaged over both ends of the bilayer plate-type system which could be defined as

4 $$\langle {T}_{Z}^{c}\rangle =({{\int }_{0}^{2\pi }{\int }_{0}^{D/2}Pdrd\varphi |}_{z=H}+{{\int }_{0}^{2\pi }{\int }_{0}^{D/2}Pdrd\varphi |}_{z=0})/\pi {(D/2)}^{2}$$

and ΔV is the volume change between the two plates, which could be written in the following form:

5 $${\rm{\Delta }}V=\pi {(D/2)}^{2}({{\int }_{0}^{2\pi }{\int }_{0}^{D/2}wdrd\varphi |}_{z=H}-{{\int }_{0}^{2\pi }{\int }_{0}^{D/2}wdrd\varphi |}_{z=0})$$

The calculated effective bulk modulus, shown as the blue hollow box curve in Fig. 3A, suggests that two bands with negative mass densities in the ranges below 358 Hz and 1050–1460 Hz were produced, while two bands with negative bulk modulus in the ranges of 856–1010 Hz and above 1508 Hz were realized, however, no double negative band with simultaneously negative effective mass density and bulk modulus was created. Through the calculation of the effective parameters, shown in Fig. 3A, with sound absorption coefficients, shown in Fig. 1B as the red solid curve with three obvious peaks denoted as A, it is evident that the effective mass densities become zero at the first absorption peak frequency, and the effective bulk modulus becomes zero at the second absorption peak frequency, while both the effective mass densities and bulk modulus become zero at the third absorption peak frequency. This means that all three absorption peaks result from the zero effective parameters (the mass density or the bulk modulus). Furthermore, in order to reveal the mechanism of the negative effective parameters, vibration displacement distributions of the three points P1, P2 and P3 shown in Fig. 3A are plotted in Fig. 3C–E, respectively. Herein, P1 is the upper edge of the first negative bulk modulus band (the first cyan shaded area), P2 is the lower edge of the second negative mass density band (the second yellow shaded area), and P3 is the frequency in which both the mass density and the bulk modulus are equal to zero. At point P1, the mass disk drives the front plate to produce the anti-resonance, and the back plate to produce a weak resonance. The two plates vibrate out-of-phase, resulting in a negative effective bulk modulus being produced by the whole structure. The resonance (or anti-resonance) shows that the direction of the plate deviates from the equilibrium position in the same (or, conversely, the opposite) direction as the propagation direction of the sound wave. At point P2, the mass disk drives the front plate to produce anti-resonance and the back plate to produce a weak anti-resonance. The two plates vibrate in-phase and generate anti-resonances with the incident sound wave as a whole, resulting in a negative mass density being produced by the entire structure. At point P3, the front plate produces a very weak anti-resonance, while the back plate produces an obvious resonance, and the two plates vibrate out-of-phase, resulting in a negative effective modulus (near zero) being produced by the entire structure. In other words, the physical mechanism of the negative mass density for these bilayer plate-type systems entails the two plates vibrating in-phase with each other (in an even motion), and vibrating out-of-phase (in anti-resonance) as a whole against the incident sound wave; while the negative bulk modulus is as a result of the out-of-phase vibration between the two plates (an uneven motion).

From Fig. 3C and D, it can be seen that at point P1 with negative bulk modulus and point P2 with negative mass density, the front plate vibrates out-of-phase with the incident sound wave, that is to say, the vibration modes at these points are similar. However, the vibration of the back plate shifts from resonant at point P1 to anti-resonant at point P2, and the effective parameters of the whole system are adjusted by the changing parameters of the back plate. Therefore, to obtain a simultaneous double negative band, the material parameters of the back plate were adjusted to be the same as the front plate, so that the Young’s modulus, Poisson’s ratio and mass density of the back plate were set at (2.3 + 0.23i) GPa, 0.37, and 1000 kg/m^3^, respectively. In addition, the mass disk was adjusted to be a steel plate with a diameter, thickness and total weight of 10 mm, of 1 mm and of 400 mg, respectively. This is denoted as model-3. The calculated dynamic effective mass density and bulk modulus is shown in Fig. 3B, which indicates that, in addition to single negative bands, a double negative band with a simultaneously negative effective mass density and bulk modulus was successfully obtained in the range of 494~586 Hz. Realizing the double negative effective parameters requires the adjustment of the monopole resonance and dipole resonance frequency bands according to the structure symmetry, which are only available for sub-wavelength local resonance structures. In these structures, the plate’s radius *R* should satisfy the condition that ${c}_{0}/f > 2R$, wherein ${c}_{0}$ and $f$ are sound speed in air and working frequency, respectively^12^.

## Acoustic Metasurfaces with Continuous Phase Shifts Covering a 2π Span

Since the air cavity usually required in acoustic metasurfaces can be formed between the layers in the bilayer system, the designed structures were expected to realize the free manipulation of phase shifts and other extraordinary capabilities of acoustic metasurfaces. In this section, we discuss the attainment of the free reflection phase shifts with a 2π span with the proposed bilayer structure, through the accurate adjustment of the geometry or material parameters. In order to achieve the acoustic metasurface-based cloak, the physical picture and working principle entail adjusting the reflection phase shift to compensate for the angle difference between the inherent scatting and the expected reflection phases, thus ensuring that the sound wave be reflected along the expected direction. Should this phase compensation be required to cover a 2π span, free manipulation of the phase shifts is required as these are critical for further applications.

**Figure 4:**
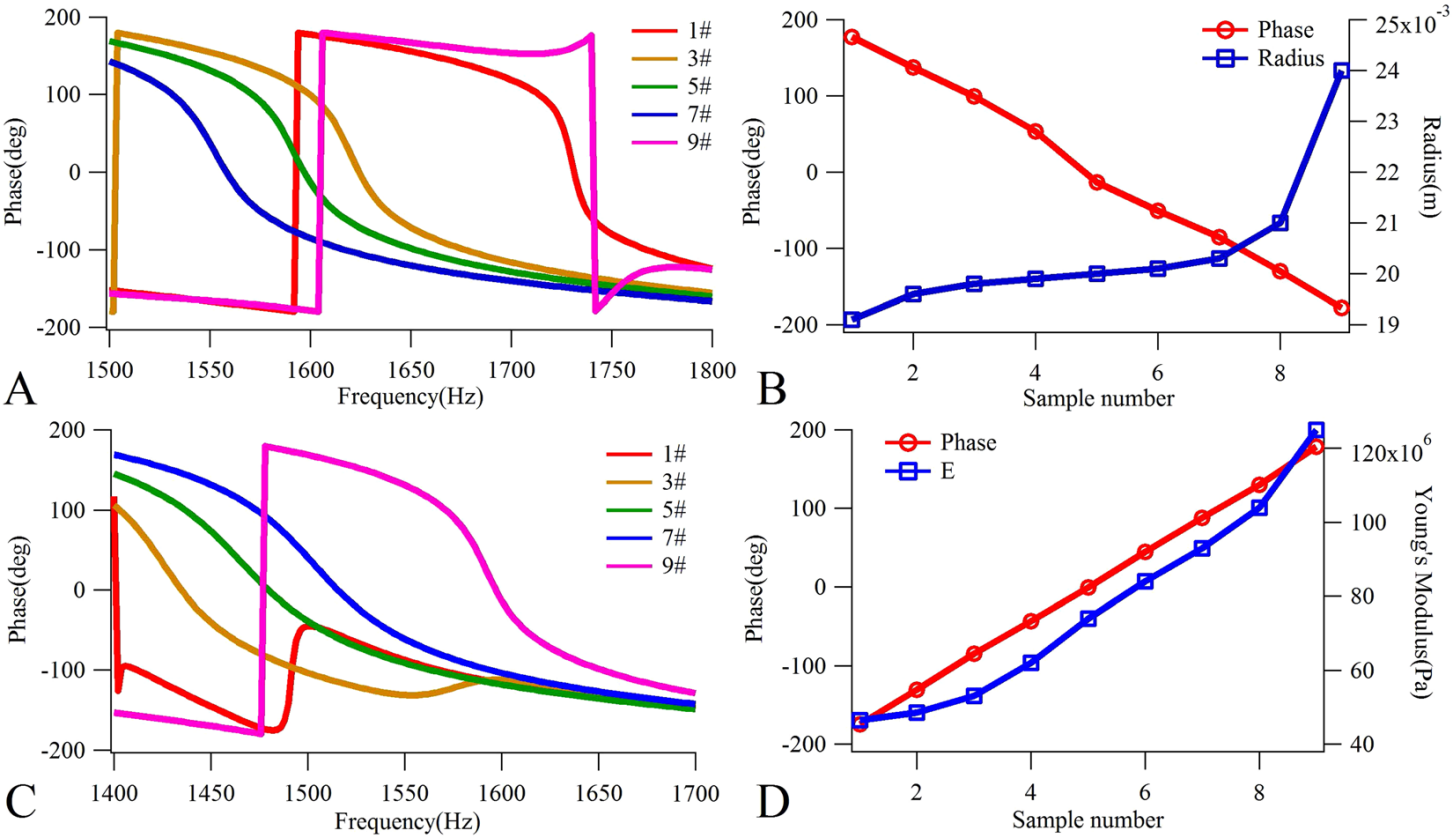
The reflection phases and continuous phase shifts of the designed acoustic metasurface structures. (**A**) The reflection phases of the designed samples with π, π/2, 0, and −π/2 phase at 1600 Hz. (**B**) The continuous phase shifts covering a 2π range in different plate radius at 1600 Hz. (**C**) The reflection phases of the designed samples with −π, −π/2, 0, and π/2 phase at 1480 Hz. (**D**) The continuous phase shifts covering a 2π range in different Young’s modulus (E) of front plate at 1480 Hz.

To achieve the above-mentioned continuous phase shifts covering a 2π range, two parameters adjustments were employed by changing the plate radius and the Young’s modulus of the front plate. Firstly, the material parameters were kept unchanged for all parts of the structure by changing the radius of the thin plate to adjust phase shifts. The Young’s modulus, Poisson’s ratio and mass density of the front plate were set at 125 MPa, 0.37 and 1000 kg/m^3^, respectively, and those of the back plate were set at 4 GPa, 0.28 and 1000 kg/m^3^, respectively. Meanwhile, the geometric and material parameters of the mass disk were kept the same as those of the model-1. Nine cells, labeled 1# to 9#, were designed to provide phase shifts ranging from π to −π with a step of π/4 at 1600 Hz. The phase profiles of 1#, 3#, 5#, 7# and 9# cells are shown in Fig. 4A. All structural and material parameters remained identical, with the exception of the radiuses of the plate and the air layer. The phases are plotted as a function of the radius of the plate in Fig. 4B, in which it can be seen that the phases decrease from π to −π as the plate radius increases from 19 mm to 24 mm. When material parameters remain unchanged, changes in the unit radius could cause a shift in the resonant frequency. Since the designed units were resonant-based structures, the phase jump from −π to π shifted with the resonant frequency, which the result that the reflected phase changed with the different unit radius to realize the regulation of phase compensation values. As can be seen from the phase profile in Fig. 4A, in order to ensure that the changes in radiuses of the plates remain as small as possible, the operating frequency can be selected within a range of approximately 1500 to 1600 Hz, wherein an arbitrarily integer value, 1600 Hz, is selected from this range. Reducing the radiuses of the plates could increase the control frequency, however, it is difficult to decrease the operating frequency to a range below 1500 Hz by changing the plate size when the material parameters remain constant. As can be seen in Fig. 4A, when the phase profile changed as the plates’ radiuses increased to 24 mm, the phase value failed to completely cover a 2π range. Therefore, the radiuses of the plates were kept as a constant of 20 mm, and the phase shifts adjusted by changing the Young’s modulus of the front plate, while the geometric and material parameters of the back plate and mass were kept the same as those in Fig. 4A. Similarly, the nine cells provided phase shifts ranging from −π to π with a step of π/4 at 1480 Hz, and the phase profiles of 1#, 3#, 5#, 7# and 9# cells are shown in Fig. 4C. The phases of these nine cells are further plotted in Fig. 4D as a function of Young’s modulus of the front plate, in which it can be seen that the phases increased from −π to π with the Young’s modulus of the front plate increasing from 46.4 MPa to 125 MPa. These results suggest that both these parameter adjusting schemes are able to realize continuous phase shifts covering a 2π range. Since the free manipulation of phase shifts offers great flexibility in the design of nonparaxial acoustic self-accelerating beams with arbitrary trajectories, the proposed metasurfaces may contribute to a variety of applications, including imaging, focus, cloak and other acoustic functional devices in acoustic engineering in which the manipulation of amplitudes and phases of wavefronts are required.

## Phase-shift-based Acoustic Cloak

**Figure 5:**
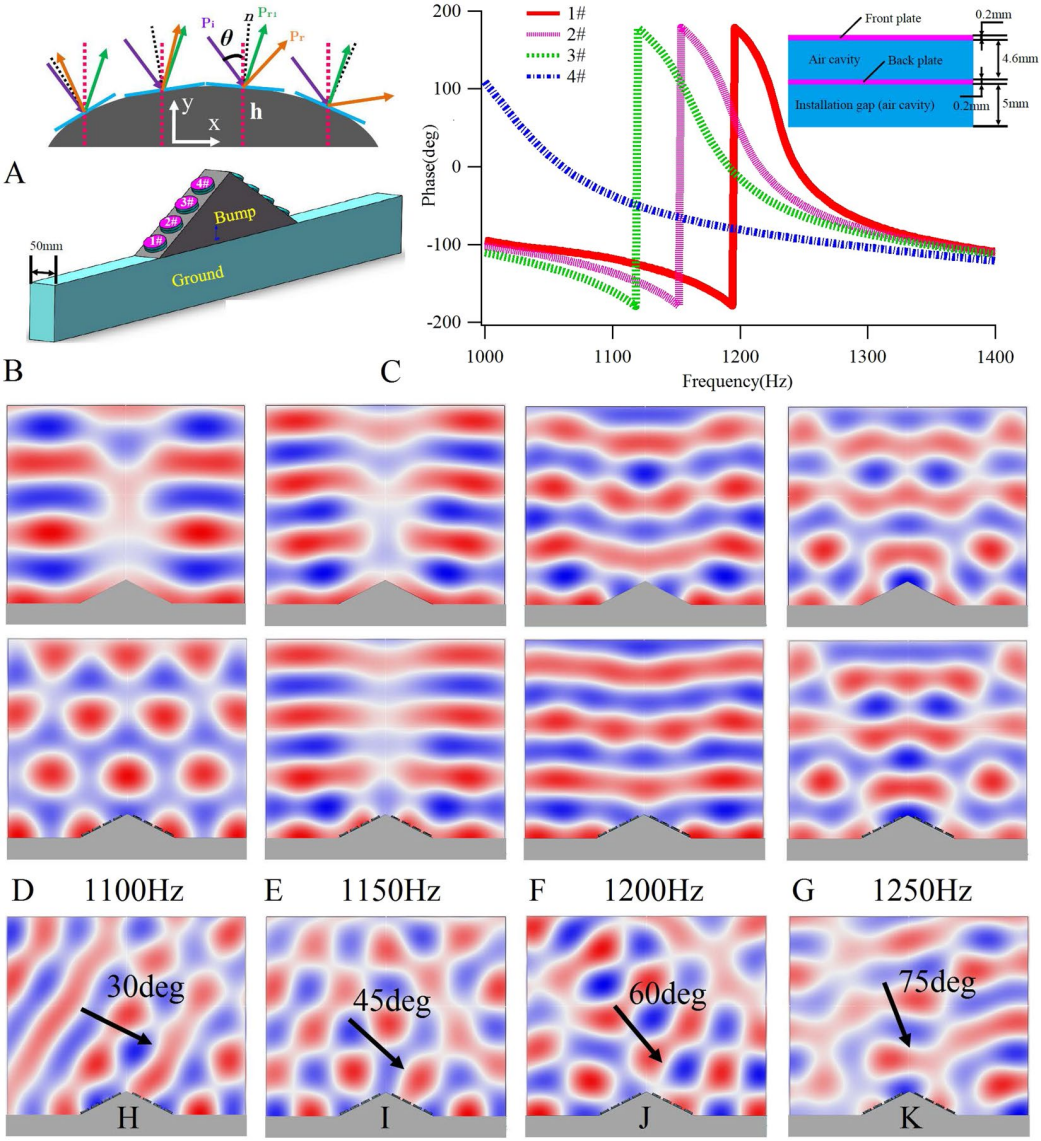
The working principle of the cloak, the designed 3D cloak structure, reflection phases of the designed unit cells, and the pressure field distributions. (**A**) The schematic for explaining the working principle of acoustic cloak. (**B**) The designed 3D cloak structure. (**C**) Reflection phases of the designed cells with pre-designed phase shifts at 1200 Hz, and the detailed structure and geometric parameters of the unit cell shown in inset. The pressure field distributions when an incoming sound plane wave perpendicularly (90 deg) illuminates the bare bump (up row) and the cloaked bump (down row) at 1100 Hz (**D**), 1150 Hz (**E**), 1200 Hz (**F**) and 1250 Hz (**G**). The pressure field distributions when an oblique incoming sound plane wave illuminates the cloaked bump at 1200 Hz with 30 deg (**H**), 45 deg (**I**), 60 deg (**J**) and 75 deg (**K**), wherein the black arrows denote the sound incident directions.

Acoustic cloak is an important application topic for acoustic metamaterials. In this section, we aim to realize the cloak through the proposed bilayer plate-type acoustic metasurface structures. A simple schematic to explain the working principle of the cloak is shown in Fig. 5A, where *P*_*i*_ is the incident wave (violet solid arrow), *P*_*r*_ is the inherent scattering wave (orange solid arrow), *P*_*r*1_ is the expected reflection wave (blue solid arrow), *n* denotes the normal vector direction (black dot line), and *θ* is the incident angle. If the wave is expected to be reflected along the *P*_*r*1_, rather than the *P*_*r*_ direction, a 2*θ* phase compensation is necessary. The required phase shifts at different positions on the surface change with the different *h*, and the incident angle *θ* may cover a 0~π range. Therefore, the allowed phase shift is required to cover a 2π range in order to achieve the free manipulation of the reflection waves into arbitrary directions. As shown in Fig. 4, the designed bilayer plate-type acoustic metasurfaces were able to provide phase compensations covering a 2π range, which, therefore, makes them suitable for the design of the acoustic cloaks. We considered an isosceles triangle-shaped object for cloaking, with a bottom edge length and height of 480 mm and 120 mm, respectively. A 3D acoustic cloak (Fig. 5B) was designed with an operating frequency of 1200 Hz, and four cells, labeled as 1# to 8#, were arranged on two slopes of the triangular object. Due to the symmetry, only the phase compensations of four cells on either the left or right side required designing. The four cells on the left side were labeled as 1# to 4# from left to right, wherein the height between the central point of the front plate and the ground was ${h}_{i}$, and the corresponding phase compensation of each cell was solved by means of the following relation:

6 $${\delta }_{i}=\pi -2{k}_{0}{h}_{i}\,\cos \,\theta $$

in which ${k}_{0}=2\pi /\lambda$ is the wave vector in air, and $\lambda$ is the manipulation wavelength.

The heights of the four designed cells were ${h}_{1}$ = 24.5 mm, ${h}_{2}$ = 51.3 mm, ${h}_{3}$ = 78.1 mm, and ${h}_{4}$ = 104.9 mm, and the corresponding phase compensations were ${\delta }_{1}$ = 2.067 rad, ${\delta }_{2}$ = 0.89 rad, ${\delta }_{3}$ = −0.29 rad, and ${\delta }_{4}$ = −1.47 rad, respectively. For simplification, a 3D bilayer plate-type structure without mass was employed, wherein the radius of the plate was 20 mm, the thicknesses of both the front and back plates was 0.2 mm, the distance between the layers was 4.6 mm, and the installation gap between the back plate and the triangular object was 5 mm. The material parameters of the back plate were kept unchanged, while the Young’s modulus of the front plate was adjusted to accommodate the reflection phase shifts. The Young’s modulus, Poisson’s ratio and mass density of the back plate were (4 + 0.2i) GPa, 0.28, and 1000 kg/m^3^, respectively, while the Young’s modulus of the front plate was (842 + 42.1i) MPa for 1# and 8#, (798 + 39.9i) MPa for 2# and 7#, (720 + 36i) MPa for 3# and 6#, and (260 + 13i) MPa for 4# and 5#, respectively, and the identical Poisson’s ratio and mass density of all cells were 0.37 and 1000 kg/m^3^, respectively. The phases of the four cells are shown in Fig. 5C. It should be noted that, in order to easily ensure the expected phase compensations, different Young’s moduli of the front plates were employed to fulfil different phase compensations in the cloak design. Although these values are not the singular values and are located in the reasonable ranges for polyethylene and other polymer materials, these material parameters should be somewhat different from those existing in nature. In practical applications, different phase compensations and operating frequencies can, therefore, be designed and further realized by precisely changing the geometric parameters (thickness or radius) of the plates after the material parameters have been determined. In this study, according to the designed cell structures, two calculation models were established to include a model with a bare triangular object and a model with a cloaked triangular object. The air domain above the structure was set as the background pressure field, which is equivalent to the planar sound wave illuminating downwards to the structure. The boundaries of the air domain were set as perfect matching layers to eliminate the boundary reflection effect. The sound pressure distributions are shown in Fig. 5D–G for the perpendicular planar sound wave and in Fig. 5H–K for the oblique illumination of the structures. These results suggest that in the narrow range around 1200 Hz, the sound pressure distributions of the cloaked structure can keep the planar sound wave field stable, while the scatter over the bare structure is obvious, thus confirming that the designed acoustic metasurface structure facilitates the cloak’s performance at the designed frequency. Moreover, the working bandwidth of the 3D plate-type metasurface structure is relatively narrow, suggesting that this structure can only exhibit the cloak effect in a narrow band around a design frequency for a perpendicular incident planar sound wave.

## Broadband Absorption-based Omnidirectional Acoustical Dark Skin

**Figure 6:**
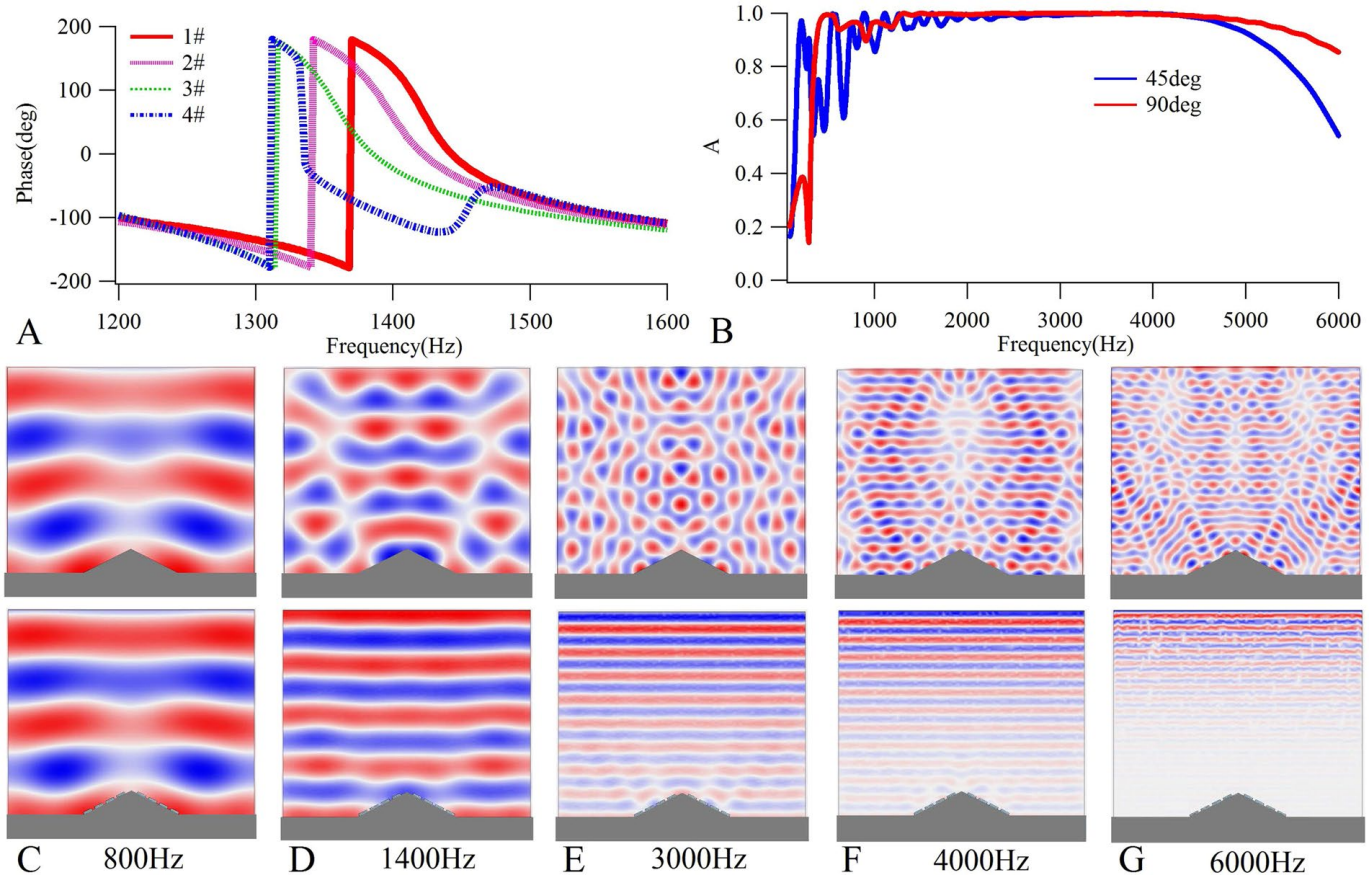
The reflection phases of the designed unit cells, sound absorption coefficients of the whole structure covered by the dark skin and pressure field distributions. (**A**) The reflection phases of the designed cells with pre-designed phase shifts at 1400 Hz. (**B**) The sound absorption coefficients of the whole dark skin covered structure when an incoming sound plane wave perpendicularly (90 deg) and obliquely (45 deg) illuminate the structure. Pressure field distributions when an incoming sound plane wave illuminate bare object (up row) and dark skin-covered object (down row) at 800 Hz (**C**), 1400 Hz (**D**), 3000 Hz (**E**), 4000 Hz (**F**), and 6000 Hz (**G**).

From the results shown in Fig. 5, it is evident that the working bandwidth of the phase-shift-based cloak is very narrow, and that the cloak performance will vanish when the sound waves obliquely illuminate the structure. Therefore, to achieve a broadband omnidirectional hidden performance, we propose a 2D broadband acoustical dark skin by employing a strong absorption concept. A 2D version of the isosceles triangle object shown in Fig. 5B was chosen as the object of stealth, and an acoustical dark skin with four cells on two slopes of the triangle were designed, for which the operating frequency was 1400 Hz. The heights of the four designed cells were ${h}_{1}$ = 15.5 mm, ${h}_{2}$ = 42.3 mm, ${h}_{3}$ = 69.2 mm, and ${h}_{4}$ = 96 mm, and the corresponding phase compensations were ${\delta }_{1}$ = 2.35 rad, ${\delta }_{2}$ = 0.972 rad, ${\delta }_{3}$ = 0.408 rad, and ${\delta }_{4}$ = −1.78 rad, respectively. The width of the plate was 40 mm, while the other geometric parameters in the thickness direction were the same as those shown in the inset of Fig. 5C. The material parameters of the back plate remained identical to those in Fig. 5, however the Young’s modulus of the front plate was adjusted to accommodate the reflection phase shifts. The Young’s modulus of the front plate was (518 + 25.9i) MPa for 1# and 8#, (464 + 23.2i) MPa for 2# and 7#, (100 + 5i) MPa for 3# and 6#, and (69 + 3.45i) MPa for 4# and 5#, respectively, while the Poisson’s ratio and mass density were the same with those in Fig. 5. The phases of the four cells are shown in Fig. 6A. We also calculated the absorption coefficients of the whole structure covered by the dark skin with different sound incident directions plotted in Fig. 6B. These suggest that, in the case of the 90 degree incident angle, in the broadband frequency range above 500 Hz, the absorption coefficients of the structure are always higher than 0.99 in the range of 1300~4500 Hz, and even at frequencies above 4500 Hz the absorptions are always higher than 0.85. The sound pressure distributions for the structure, either without dark skin (up row) or with dark skin (down row), are shown in Fig. 6C–G. It can be seen that, in the broadband range of 800~6000 Hz, the sound pressure distributions of the dark skin-covered structure could maintain the planar sound wave field, while the scattering of the bare triangle structure was obvious, thus confirming that the designed acoustic metasurface structure can provide broadband stealth performance. As the thickness of the designed structure was only 5 mm, the equivalent to 1/49 of the operating wavelength, the subwavelength level was very high. Moreover, in the design of such an acoustical dark skin, only eight cells are used in all, making it convenient for a wide range of applications.

**Figure 7:**
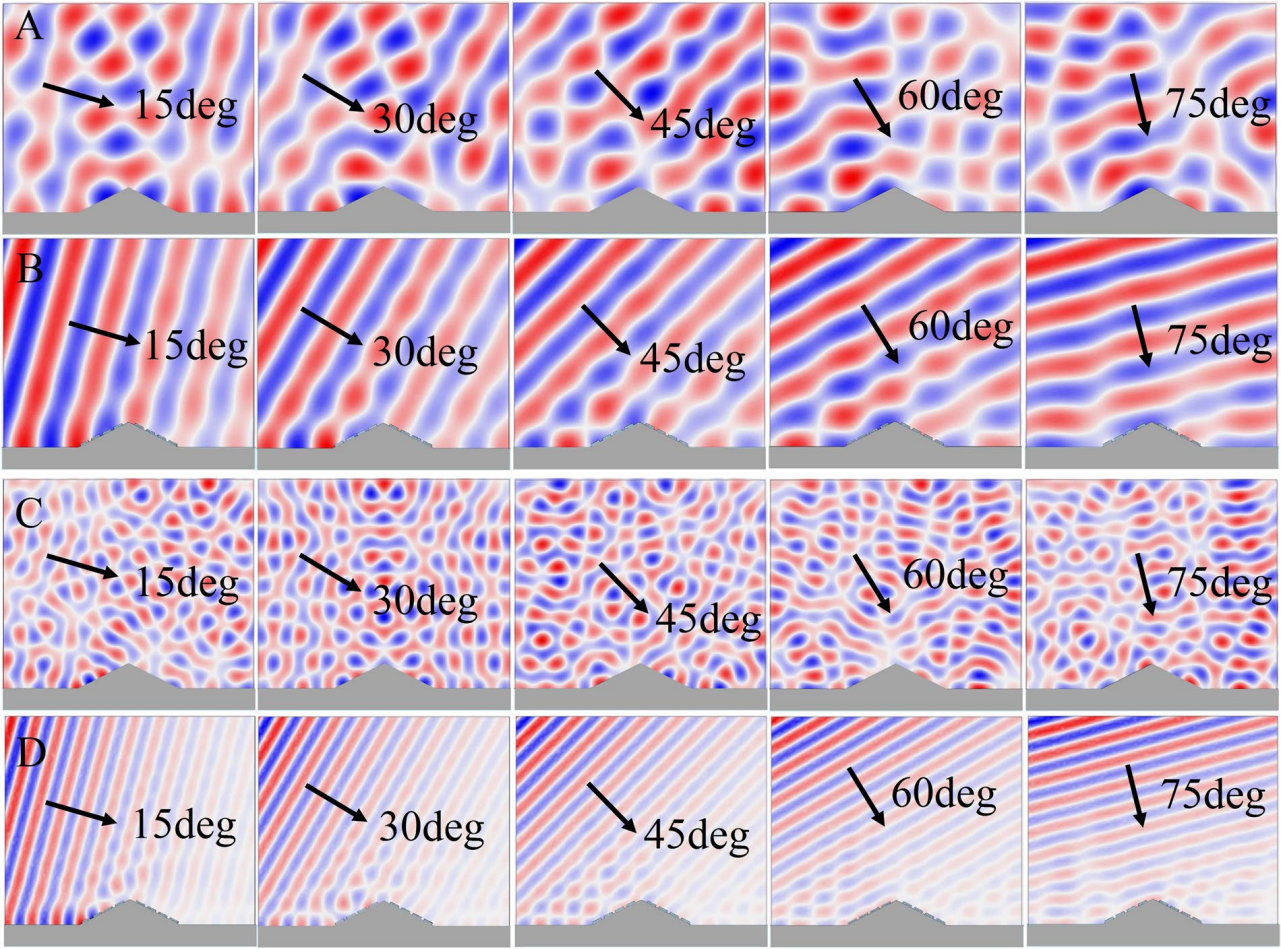
The pressure field distributions for the oblique incident wave. The pressure field distributions when an oblique incoming sound plane wave illuminates the bare object (**A**) for 1400 Hz, and (**C**) for 3000 Hz) and the dark skin-covered object (**B**) for 1400 Hz, and (**D)** for 3000 Hz) with 15 deg, 30 deg, 45 deg, 60 deg and 75 deg, wherein the black arrows denote the sound incident directions.

In addition to the normal incidence condition, the oblique incidence was also considered in this study. The pressure field distributions, in which an oblique incoming sound plane wave illuminates both the bare object and the dark skin-covered object from different angles, are shown in Fig. 7. These figures indicated that under omnidirectional incident directions in the broadband range, the sound pressure distributions of the dark skin-covered structure maintained a fine planar sound wave field, while the scatter of the bare structure was obvious, confirming that the designed acoustic metasurface structure delivers broadband omnidirectional stealth performance. It must be emphasized that the phase shift of the metasurface was determined by Eq. (6), which is obviously dispersive, and, therefore, linear phase shifts provided by cells are necessary to realize the broadband cloak performance. However, as shown in Fig. 6A, even in the range of 1200~1600 Hz, the phase shifts of these four cells were obviously nonlinear. Consequently, it can be deduced that the broadband stealth performance is not determined by the phase shift theory of metasurface. In fact, the sound absorption results in Fig. 6B suggest that, since almost all the incident sound waves could be absorbed by the dark skin-covered structure, the scatter waves could be effectively eliminated, followed by a broadband stealth effect. In addition, because the structure sizes of the used unit cells were subwavelength and the acoustical properties are insensitive to the incident sound direction in the low frequency ranges, the sound absorption coefficients of the dark skin were also very high when the sound obliquely illuminated the structure, followed by the omnidirectional stealth performance. As the stealth performance of this structure is determined by its strong sound absorption, rather than the phase shifts at dispersive frequencies, it demonstrates a hidden acoustical design strategy based on strong absorptions for broadband and omnidirectional stealth. Only the two-dimensional cloak in XOY plane is considered herein, however, a condition of realizing the broadband acoustical dark skin is that the size in the Z direction was large enough to ensure that any influences caused by the manipulation of boundary conditions of the plate would be negligible.

## Conclusions

In this paper, we have proposed a general design concept for acoustic functional devices by introducing a synergetic behavior between the resonance and anti-resonance features, in which each component provides a pre-defined purpose. Furthermore, on the basis of such a synergetic coupling design concept, a bilayer plate-type acoustic metasurface was proposed, and the designed bilayer samples were fabricated in order to experimentally measure their absorption, reflection and transmission coefficients. The results confirmed an excellent agreement between the experimental results and the calculated results, both of which suggest that the proposed bilayer plate-type structure could realize strong, and even perfect, sound absorption. In addition, the effective parameters of the designed structures were solved through effective media theory, and double negative bands with a simultaneously negative effective mass density and bulk modulus were obtained. Moreover, since the design of the proposed bilayer structure makes it possible to form the usually required air cavity for an acoustic metasurface between the layers, it was possible to obtain continuous phase shifts almost completely covering a 2π range, by adjusting either the radius or Young’s modulus of the plates. Consequently, a 3D narrow band acoustic cloak was designed based on this concept of the phase shifts. In addition, a 2D acoustical dark skin, with ultrathin sizes of 1/49-wavelength in the plate thickness direction and covering frequencies from 800 to 6000 Hz was demonstrated through the realization of the broadband omnidirectional stealth performance and the concept of strong absorption. The proposed design concept and the bilayer plate-type metasurface structures therefore offer widespread and significant application values in areas of acoustic engineering such as strong sound attenuations, filtering, superlens, imaging, cloak and extraordinary wave steering, in which features such as a perfect absorption, double negative parameters or continuous phase shifts covering a 2π range are needed.

## Methods

### Experiment measurements

The experiment samples consisted of a rigid nylon plate (the back layer), and a soft polyethylene terephthalate (PET) plate (the front layer) decked with a rigid additional mass disk with a 3 mm radius. This weight was adjusted with the addition of 100 mg and 200 mg plasticine on the disk, for the first and second samples, respectively. In assembly, these two plates, each with an identical 20 mm radius and 0.2 mm thickness, were fixed at a distance of 20 mm apart by a rigid polylactice acid (PLA) frame (Fig. 1A). The sound absorptions, reflections and transmissions shown in Fig. 1B were measured by a Brüel & Kjær Type-4206 impedance tube kit^32^.

### Numerical simulations

The simulations were performed with the commercial finite element analysis solver, Comsol Multiphysics R4.3a. In the simulations of the unit cells, a two-dimensional axisymmetric module was employed to reduce the calculation cost, and the frames were modeled as clamped boundary conditions at the edges of plates. Plane wave radiation boundary conditions were set at the input and output planes of the air domains in the simulation models of the unit cells.
